# Editorial: Recent Advances in Electroreception and Electrogeneration

**DOI:** 10.3389/fnint.2021.668677

**Published:** 2021-03-11

**Authors:** Maurice J. Chacron, Michael R. Markham

**Affiliations:** ^1^Department of Physiology, McGill University, Montreal, QC, Canada; ^2^Department of Biology, University of Oklahoma, Norman, OK, United States

**Keywords:** neuroscience, weakly electric fishes, neuroethology, electrogeneration, electroreception

The study of fish that generate electric fields around their bodies in order to interact with their environment continues to generate intense interest in the research community. While it has been known since ancient times that some fish can generate electricity (Gaillard, 1923), the fact that some of these animals use the resulting electric field to detect objects and communicate with conspecifics has been discovered far more recently (Lismann and Machin, 1958; Lissmann, 1958). These animals have led to key discoveries in cholinergic transmission as well as the development of modern electrophysiology (Wu, 1984). Today, studies of electric fishes continue to generate important discoveries. This issue contains 11 articles (3 review and 8 original research) that highlight the diversity of recent research progress using electric fishes that range from studying the effects of changes in hormonal levels and the natural environment on electrosensory behaviors, to understanding how the brains of electric fishes process natural stimuli. This issue not only reviews the contributions that studies of electric fish have made toward understanding key problems in sensory processing (e.g., how does the brain distinguish sensory input that is self vs. externally-generated; Heiligenberg, 1991), but also provides novel functions for some of behavioral responses displayed by electric fishes such as natural electrocommunication stimuli as well as the jamming avoidance response that has been studied for over 40 years (Heiligenberg, 1991; Zakon et al., 2002).

Fukutomi and Carlson provide a very nice historical review of the contribution of mormyrid weakly electric fish toward understanding the function of the corollary discharge in distinguishing the sensory consequences of self vs. externally generated stimuli. This is a general problem that every animal must solve in order to successfully interact with its environment and common mechanisms have emerged across species (Cullen, 2004). It has furthermore become clear that the brain is not a simple input-output device but that the brain's internal state strongly influences perception and behavior (Hurley et al., 2004). Toward that end, Marquez and Chacron investigate the effects of serotonergic neuromodulation on sensory processing.

In general, processing of sensory input by the brain is achieved through a combination of neural circuits as well as ion channels embedded in the cell membrane. Toward the former end, Hofmann and Chacron review recent findings on the role of descending pathways from higher brain areas to more peripheral ones, which are found ubiquitously in the CNS of vertebrates (Cajal, 1909), on actually generating neural responses to sensory input. Toward the latter end, Motipally et al. investigate the expression of sodium channels across parallel sensory maps, with functional implications for differential coding of behaviorally relevant stimulus features by bursts (i.e., packets of action potentials followed by quiescence). Such bursts have been shown to reliably encode electro-communication stimuli that occur during agonistic encounters (Marsat et al., 2009). Metzen provides a review of how natural electrocommunication stimuli are processed across successive brain areas in order to give rise to perception and behavioral responses. Interestingly, Field et al. suggest an important new function for natural electro-communication stimuli toward actually avoiding the deleterious effects of jamming stimuli, a function that was previously thought to be carried out by the jamming avoidance response. Specifically, when two fish are located within close proximity of one another, interference between their electric fields can create a jamming signal that interferes with the animal's ability to electrolocate other relevant stimuli such as prey or object boundaries (Ramcharitar et al., 2005). The animal solves this problem by changing its EOD characteristics in order to increase the frequency content of the jamming signal away from that of other electrosensory stimuli that it must detect (Heiligenberg, 1991). This so-called “classical function” of the jamming avoidance response is also being questioned by Fortune et al. in their investigation of how evolutionary loss of vision affects electroreception in different species of glass knifefishes and, in particular, demonstrate lack of jamming avoidance responses despite social conditions that would normally trigger such behavior based on previous studies.

Interestingly, Fortune et al. also demonstrate that blind electric fish display more territoriality and dominance than their seeing cousins. Raab et al. provide some critical field data as to dominance and exploration behavior. Such field data will no doubt be key toward guiding future studies as to how these animals actively acquire information about their environments in natural settings. A study by Yu et al. investigates how an important aspect of electrosensory stimuli experienced by electric fish during social interactions, namely contrast, strongly depends on the relative distance and orientation between animals. In particular, Yu et al. reveal that contrast routinely reaches much higher values than are typically used in laboratory settings (see e.g., Marsat et al., 2009). An important aspect of electrosensory research is that laboratory studies of sensory processing have often focused on recording from immobilized animals. Future studies are needed where recordings are instead obtained from freely moving animals in order to better mimic natural conditions where animals actively sense their environment (Schroeder et al., 2010). Such approaches have recently been applied to record neural activity from forebrain in weakly electric fish (Fotowat et al., 2019) and are becoming increasingly common in aquatic species (Cohen et al., 2019).

In addition to research on the sensory processing of electric signals during social encounters, it is also critical to investigate the central networks and peripheral mechanisms that regulate electric signaling in social contexts to fully understand the social communication functions of electric signals. Toward this end, Pouso et al. report, for the first time in electric fish, that distinct classes of vasotocin neurons within the pre-optic area are preferentially activated by social stimuli in courting males, and that these changes are associated with the production of electric courtship signals as well as locomotor activity related to courtship.

Finally, it is important to remember that the most widely recognized electric fish, the Electric Eel, not only uses weak electric fields to sense the environment but also uses strong electric fields as a weapon to stun and capture prey. Catania reviews recent advances showing how electric eels adapt the characteristics of their strongly electric fields for different behavioral contexts ranging from stunning prey to self-defense. Interestingly, electric eels also use their strong electric field to actually track prey, which was until recently thought to be achieved exclusively through the weak electric field.

Taken together, the manuscripts in this collection help to illustrate the many historical advances and current progress that research with electric fish contributes to neuroethology and integrative neurobiology. This collection also serves to highlight the vast range of unresolved questions yet to be experimentally addressed.

## Author Contributions

MC and MM wrote the manuscript. All authors contributed to the article and approved the submitted version.

## Conflict of Interest

The authors declare that the research was conducted in the absence of any commercial or financial relationships that could be construed as a potential conflict of interest.
